# Functional domains of *Acinetobacter* bacteriophage tail fibers

**DOI:** 10.3389/fmicb.2024.1230997

**Published:** 2024-04-16

**Authors:** Danielle L. Peters, Francis Gaudreault, Wangxue Chen

**Affiliations:** ^1^Human Health Therapeutics (HHT) Research Center, National Research Council Canada, Ottawa, ON, Canada; ^2^HHT Research Center, NRC, Montreal, QC, Canada; ^3^Department of Biology, Brock University, St. Catharines, ON, Canada

**Keywords:** *Acinetobacter*, bacteriophage, protein domains, depolymerase, pectin lyase, tail spike, antimicrobial resistance

## Abstract

A rapid increase in antimicrobial resistant bacterial infections around the world is causing a global health crisis. The Gram-negative bacterium *Acinetobacter baumannii* is categorized as a Priority 1 pathogen for research and development of new antimicrobials by the World Health Organization due to its numerous intrinsic antibiotic resistance mechanisms and ability to quickly acquire new resistance determinants. Specialized phage enzymes, called depolymerases, degrade the bacterial capsule polysaccharide layer and show therapeutic potential by sensitizing the bacterium to phages, select antibiotics, and serum killing. The functional domains responsible for the capsule degradation activity are often found in the tail fibers of select *A. baumannii* phages. To further explore the functional domains associated with depolymerase activity, tail-associated proteins of 71 sequenced and fully characterized phages were identified from published literature and analyzed for functional domains using InterProScan. Multisequence alignments and phylogenetic analyses were conducted on the domain groups and assessed in the context of noted halo formation or depolymerase characterization. Proteins derived from phages noted to have halo formation or a functional depolymerase, but no functional domain hits, were modeled with AlphaFold2 Multimer, and compared to other protein models using the DALI server. The domains associated with depolymerase function were pectin lyase-like (SSF51126), tailspike binding (cd20481), (Trans)glycosidases (SSF51445), and potentially SGNH hydrolases. These findings expand our knowledge on phage depolymerases, enabling researchers to better exploit these enzymes for therapeutic use in combating the antimicrobial resistance crisis.

## 1 Introduction

Antimicrobial resistance (AMR) is a major threat to human health, with watch lists created for several bacteria due to their high levels of resistance. A global delay in action against AMR, coupled with the COVID-19 pandemic, has further exacerbated the AMR crisis. The Centers for Disease Control and Prevention (CDC) published a special report in 2022 detailing a 15% increase in resistant nosocomial infections from 2019 to 2020. Of particular concern is the significant increase in carbapenem-resistant *Acinetobacter baumannii* infections, with a 78% increase between 2019 and 2020 (COVID-19: U.S. Impact on Antimicrobial Resistance Special Report 2022, 2022). Due to multidrug-resistant outbreaks involving *A. baumannii*, the World Health Organization classified it as a number one priority pathogen that is urgently in need of alternative treatment options (World Health Organization, 2021).

Novel treatment options are desperately needed to address the critical lack of therapeutics against *A. baumannii* infections. One treatment approach is the use of its evolutionary predator–bacteriophages (phages). Phages, and their biological products such as endolysins and depolymerases, are attracting renewed interest as therapeutic options due to their high specificity, potential synergy with antibiotics, unique mode of action, safety, natural abundance, and modification potential (Melo et al., 2020). The narrow host range of phages, as well as the requirement of the phages to successfully enter the cell and propagate to produce progeny phages, complicates the use of phages in therapy. Recent advancements in engineering host receptor binding proteins to expand a phage's host range have shown great success, though it does not eliminate the requirement of the phage to successfully propagate in the cell (Dams et al., 2019; Yehl et al., 2019).

The use of specialized phage enzymes, such as depolymerases or endolysins, instead of active phages could be a potential solution. There are two main groups of phage-associated enzymes responsible for the degradation of carbohydrate-containing polymers: hydrolases (EC 3) and lyases (EC 4) (Latka et al., 2017). Hydrolases target the peptidoglycan, capsular polysaccharides, or the O-antigen side chains of lipopolysaccharides by catalyzing the cleavage of O-glycosidic bonds using water. Lyases utilize β-elimination to introduce a double bond between the C4 and C5 of the non-reducing uronic acid after cleavage of the glycosidic bond between a monosaccharide and the C4 of uronic acid (Sutherland, 1999). These phage depolymerases are found as integral components of the virion structure or as soluble proteins that diffuse out following the host cell lysis (Drulis-Kawa et al., 2015). Phage depolymerases are most often identified within tail fibers or tail spikes and generally form as homotrimeric complexes.

Many groups have investigated phage-encoded depolymerases and endolysins for therapeutic use (Liu et al., 2019a; Kim et al., 2020; Khan et al., 2021; Abdelkader et al., 2022; Chen et al., 2022; Drobiazko et al., 2022). The potential therapeutic benefit of depolymerases is the removal of the capsule layer from the infecting bacterium, which exposes the bacterium to the innate immune system and thus activates serum killing. The potential use of depolymerases for therapy against *A. baumannii* has been studied and demonstrated in mouse models with a promising therapeutic effect (Liu et al., 2019a; Oliveira et al., 2019; Wang et al., 2020). In this study, we analyzed the tail fibers of published *Acinetobacter* phages and identified different functional domains present. These data will enable further research and development on these exciting enzymes for therapeutic use.

## 2 Methods

### 2.1 Analysis of *Acinetobacter* phage genomes for tail fibers

A total of 114 *Acinetobacter* phages were identified in the literature as of 22 July 2021. Phage genomes were downloaded from the NCBI database using the accession numbers presented in each article. Information on the presence or absence of halo formation was collected for each phage. All coding sequences (CDS) in the morphogenesis module of the phage genomes were translated and analyzed using the InterProScan (Jones et al., 2014; Blum et al., 2021) plugin for Geneious Prime 2021.2.2 to identify any functional domains present within the tail fibers. InterProScan ran using the following applications: conserved domains database (CDD), Gene3D, high-quality automated and manual annotation of proteins (HAMAP), protein analysis through evolutionary relationships (PANTHER), Pfam-A, PRINTS, PROSITE profiles, simple modular architecture research tool (SMART), and SUPERFAMILY (Jones et al., 2014; Blum et al., 2021).

The CDD is composed of curated protein domain and protein family models that are searched using reverse position-specific BLAST to match protein sequences with domain and family models (Yang et al., 2020). Gene3D is a comprehensive database of protein domain assignments for sequences from major sequence databases including Ensembl, UniProt, and RefSeq, where domains are directly mapped from structures in the class, architecture, topology, homology (CATH) database or predicted with a library of representative profile hidden Markov models (HMMs) derived from CATH superfamilies (Cuff et al., 2011; Lees et al., 2012). HAMAP is an automatic annotation pipeline that uses a collection of family profiles and manually curated signatures to determine protein family membership of a query protein sequence (Pedruzzi et al., 2015). PANTHER is used to classify sequences into evolutionary groupings (protein class, family, subfamily) using phylogenetic trees, and functional groupings with gene ontology terms and pathways (Mi et al., 2021). The Pfam-A database is a comprehensive collection of protein families, where each family is represented by a curated set of multiple sequence alignments and HMMs (Mistry et al., 2021). The PRINTS database is comprised of a collection of protein family ‘fingerprints' or a group of conserved motifs that provide distinctive signatures for particular protein families and structural/functional domains (Attwood et al., 2012). PROSITE profiles is a database of protein families and domains with specific signatures on constant and variable properties of proteins that can enable the formation of hypotheses about the protein's function (Sigrist et al., 2012). The SMART database is used for the identification and annotation of protein domains and the analysis of protein domain architectures using manually curated models (Letunic et al., 2021). SUPERFAMILY is a database composed of HMMs of structural protein domains that have an evolutionary relationship (Gough et al., 2001).

Functional domain groups were formed for comparison using the identified InterProScan hit results. Multisequence protein alignments were generated using Clustal Omega v.1.2.3 (Sievers and Higgins, 2018). Phylogenetic trees were built from the resulting protein sequence alignments using Randomized Axelerated Maximum Likelihood (RAxML)v.8.2.11 with the following settings: protein model GAMMA BLOSUM62, rapid bootstrapping and search for best-scoring ML tree with 100 bootstrap replicates, and parsimony random seed of 1 (Stamatakis, 2014).

Further investigation into the tertiary structure of specific proteins was completed using AlphaFold v.2.3.1 (Jumper et al., 2021) using the multimer_v3 models. The proteins were modeled as homotrimers as this is the most common oligomeric state phage depolymerases adopt (Latka et al., 2017). The models were visually inspected to ensure good structural integrity in the fold. Only the model with the highest confidence according to AlphaFold was analyzed further. The predictions were run on the Digital Research Alliance of Canada superclusters and required 300 h of A100 GPU computing time. The models were submitted to the DALI server (Holm, 2022) to compare the predicted structures to pre-existing ones. The models were made accessible in the PDB format in the Supplementary material. In addition, all abbreviations are provided in Supplementary Table 1.

## 3 Results and discussion

### 3.1 Characteristics of the phages used in the analysis

Of the 114 phages documented, a total of 43 phages had no genomic data or had poor genome assemblies and were excluded from the analysis; thus, 71 characterized and sequenced phages were identified for further study (Table 1). Based on the literature review of the viruses documented at the time, the genomes of 31 myoviruses, 33 podoviruses, and 7 siphoviruses were downloaded from the NCBI database and 94 tail fibers were identified and translated. The majority of phages (69%) were predicted to encode one tail fiber, while 20 phages (28%) encoded two tail fibers, and two phages (3%, B9 and fHyAci03) encoded three tail fibers (Table 1). Furthermore, the presence of halo formation around the phage plaques, or expression of a depolymerase, is noted in Table 1 under “Halo or depolymerase”. In total, 43 phages were identified as having halo formation or a functional depolymerase was expressed. Only three phages were explicitly described as lacking halo formation (KARL-1, TAC1, and Loki).

**Table 1:** *Acinetobacter baumannii* bacteriophages and their tail fiber proteins used in the analysis.

**Name**	**Accession**	**Length (bp)**	**Protein IDs**	**Halo or depolymerase**	**Type**	**References**
Ab 121	MT623546	102499	QMP18972.1	N/A	Myovirus	Wu et al., 2021
AB1	HM368260	45159	ADO14448.1, ADO14447.1	Yes	Myovirus	Li et al., 2012
AbTJ	MK340941	42670	QAU04146.1	Yes	Myovirus	Xu et al., 2020
IME-AB2	JX976549	43665	AFV51555.1, AFV51556.1	N/A	Myovirus	Peng et al., 2014
SH-Ab 15599	MH517022	143204	AXF41547.1	Yes	Myovirus	Hua et al., 2018
AbP2	MF346584	45373	ASJ78889.1, ASJ78888.1	Yes	Myovirus	Yang et al., 2019
Abp9	MN166083	44820	QEA11049.1	Yes	Myovirus	Jiang et al., 2020
TaPaz	MZ043613	93703	QVW53859.1, QVW53860.1	Yes	Myovirus	Shchurova et al., 2021
AbTZA1	NC_049445	168223	YP_009882247.1	N/A	Myovirus	Nir-Paz et al., 2019
AM24	KY000079	97177	APD20249.1	Yes	Myovirus	Popova et al., 2019
AP22	HE806280	46387	CCH57762.1, CCH57761.1	Yes	Myovirus	Popova et al., 2012
BS46	MN276049	94068	QEP53229.1	Yes	Myovirus	Popova et al., 2020b
DMU1	MT992243	43482	QOI69765.1	N/A	Siphovirus	Pehde et al., 2021
YMC-13-01-C62 (C62)	KJ817802	44844	AID17959.1, AID17960.1	N/A	Myovirus	Jeon et al., 2016b
YMC11/12/R1215 (R1215)	KP861231	44866	AJT61417.1, AJT61416.1	N/A	Myovirus	Jeon et al., 2016a
YMC11/12/R2315 (R2315)	KP861229	44846	AJT61314.1, AJT61315.1	N/A	Myovirus	Jeon et al., 2016a
KARL-1	MH713599	166560	YP_009881534.1, YP_009881544.1	No	Myovirus	Jansen et al., 2018
YMC13/03/R2096 (R2096)	KM672662	98170	AIW02800.1, AIW02768.1	N/A	Myovirus	Jeon et al., 2019
Ab124	MT633129	40471	QMP19165.1	N/A	Podovirus	Wu et al., 2021
AB3	KC311669	31185	AGC35305.1	Yes	Podovirus	Zhang et al., 2015
Presley	KF669658	77792	AGY48147.1	N/A	Podovirus	Farmer et al., 2013
SH-Ab 15497	MG674163	43420	AUG85465.1	N/A	Siphovirus	Hua et al., 2018
Abp1	JX658790	42185	AFV51022.1	Yes	Podovirus	Huang et al., 2013
Fri1	KR149290	41805	AKQ06854.1	Yes	Podovirus	Knirel et al., 2020
TAC1	MK170160	101770	AZF88430.1	No	Myovirus	Asif et al., 2020
IME200	KT804908	41243	ALJ97635.1	Yes	Podovirus	Liu et al., 2019b
vB_AbaM_Acibel004 (Acibel004)	KJ473422	99730	AHY26763.1, AHY26738.1	N/A	Myovirus	Merabishvili et al., 2014
vB_AbaM_B09_Aci01-1 (Aci01-1)	MH800198	103628	AYD85523.1	N/A	Myovirus	Essoh et al., 2019
vB_AbaM_B09_Aci02-2 (Aci02-2)	MH800199	104354	AYD85686.1	N/A	Myovirus	Essoh et al., 2019
vB_AbaM_B09_Aci05 (Aci05)	MH746814	102789	AYD82311.1	N/A	Myovirus	Essoh et al., 2019
vB_AbaM_B9 (B9)	MH133207	93641	AWD93202.1, AWD93211.1, AWD93192.1	Yes	Myovirus	Oliveira et al., 2018
vB_AbaM_IME285 (IME285)	MH853786	45063	AYP68900.1	Yes	Myovirus	Wang et al., 2020
vB_AbaM_ME3 (ME3)	KU935715	234900	AND75265.1, AND75267.1	N/A	Myovirus	Buttimer et al., 2016
vB_AbaM_PhT2 (PhT2)	MN864865	166330	QHJ75775.1	N/A	Myovirus	Kitti et al., 2014
vB_AbaP_46-62_Aci07 (Aci07)	MH800200	42330	AYD85862.1	Yes	Podovirus	Essoh et al., 2019
Petty	KF669656	40739	AGY48011.1	Yes	Podovirus	Hernandez-Morales et al., 2018
phiAB1	HQ186308	41526	ADQ12745.1	N/A	Podovirus	Li et al., 2012
phiAB6	KT339321	40570	ALA12264.1	Yes	Podovirus	Lai et al., 2016
vB_AbaP_APK32 (APK32)	MK257722	41142	AZU99395.1	Yes	Podovirus	Popova et al., 2020a
SH-Ab 15519	KY082667	40493	APD19440.1	Yes	Podovirus	Hua et al., 2018
vB_AbaP_Acibel007 (Acibel007)	KJ473423	42654	AHY26817.1	N/A	Podovirus	Merabishvili et al., 2014
vB_AbaP_APK48 (APK48)	MN294712	41105	QFG06960.1	Yes	Podovirus	Popova et al., 2020a
vB_AbaP_APK116 (APK116)	MN807295	41765	QHS01530.1	Yes	Podovirus	Popova et al., 2020a
vB_AbaP_APK2 (APK2)	MK257719	41476	AZU99242.1	Yes	Podovirus	Popova et al., 2020a
vB_AbaP_APK37 (APK37)	MK257723	41981	AZU99445.1	Yes	Podovirus	Popova et al., 2020a
vB_AbaP_APK44 (APK44)	MN604238	41461	QGK90444.1	Yes	Podovirus	Popova et al., 2020a
vB_AbaP_AS12 (AS12)	KY268295	41402	APW79830.1	Yes	Podovirus	Popova et al., 2017
vB_AbaP_APK87 (APK87)	MN604239	42402	QGK90498.1	Yes	Podovirus	Popova et al., 2020a
vB_AbaP_APK89 (APK89)	MN651570	41198	QGK90394.1	Yes	Podovirus	Popova et al., 2020a
vB_AbaP_APK93 (APK93)	MK257721	41668	AZU99342.1	Yes	Podovirus	Popova et al., 2020a
vB_AbaP_B5 (B5)	MF033349	41608	ASN73455.2	Yes	Podovirus	Oliveira et al., 2017
vB_AbaP_AS11 (AS11)	KY268296	41642	AQN32697.1	Yes	Podovirus	Popova et al., 2017
vB_AbaP_PD-6A3 (PD-6A3)	NC_028684	41563	YP_009190472.1	Yes	Podovirus	Wu et al., 2019
vB_AbaP_PD-AB9 (PD-AB9)	KT388103	40938	ALM01895.1	N/A	Podovirus	Wu et al., 2019
vB_AbaP_B09_Aci08 (Aci08)	MH763831	42067	AYD82867.1	Yes	Podovirus	Essoh et al., 2019
vB_AbaS_TRS1 (TRS1)	KX268652	40749	ANT40742.1, ANT40741.1	N/A	Siphovirus	Turner et al., 2016
vB_ApiM_fHyAci03 (fHyAci03)	MH460829	165975	AXF40726.1, AXF40736.1, AXF40808.1	N/A	Myovirus	Pulkkinen et al., 2019
vB_ApiP_P1 (P1)	MF033350	41208	ASN73504.1	Yes	Podovirus	Oliveira et al., 2017
vB_AbaP_B1 (B1)	MF033347	40879	ASN73353.1	Yes	Podovirus	Oliveira et al., 2017
WCHABP1	KY829116	45888	ARQ94727.1, ARQ94726.1	Yes	Myovirus	Zhou et al., 2018
WCHABP12	KY670595	45415	ARB06756.1, ARB06757.1	Yes	Myovirus	Zhou et al., 2018
vB_AbaP_B3 (B3)	MF033348	40598	ASN73401.1	Yes	Podovirus	Oliveira et al., 2017
vB_AbaP_D2 (D2)	MH042230	39964	AVP40472.1	Yes	Podovirus	Yuan et al., 2021
vB_AbaP_PMK34 (PMK34)	MN433707	41847	QGF20174.1	Yes	Podovirus	Abdelkader et al., 2020
vB_ApiP_P2 (P2)	MF033351	41514	ASN73558.1	Yes	Podovirus	Oliveira et al., 2017
ZZ1	HQ698922	166687	AEJ90215.1, AEJ90224.1	N/A	Myovirus	Jin et al., 2012
fEg-Aba01	MT344103	33779	QJT69876.1, QJT69875.1	Yes	Siphovirus	Badawy et al., 2020
fLi-Aba02	MT344104	35093	QJT69930.1, QJT69929.1	Yes	Siphovirus	Badawy et al., 2020
fLi-Aba03	MT344105	34931	QJT69984.1, QJT69983.1	Yes	Siphovirus	Badawy et al., 2020
phiAC-1	JX560521	43216	AFU62318.1	N/A	Myovirus	Kim et al., 2012
Loki	LN890663	41308	CUS06481.1	No	Siphovirus	Turner et al., 2017

### 3.2 Domains of the phage tail fibers

**Figure 1:**
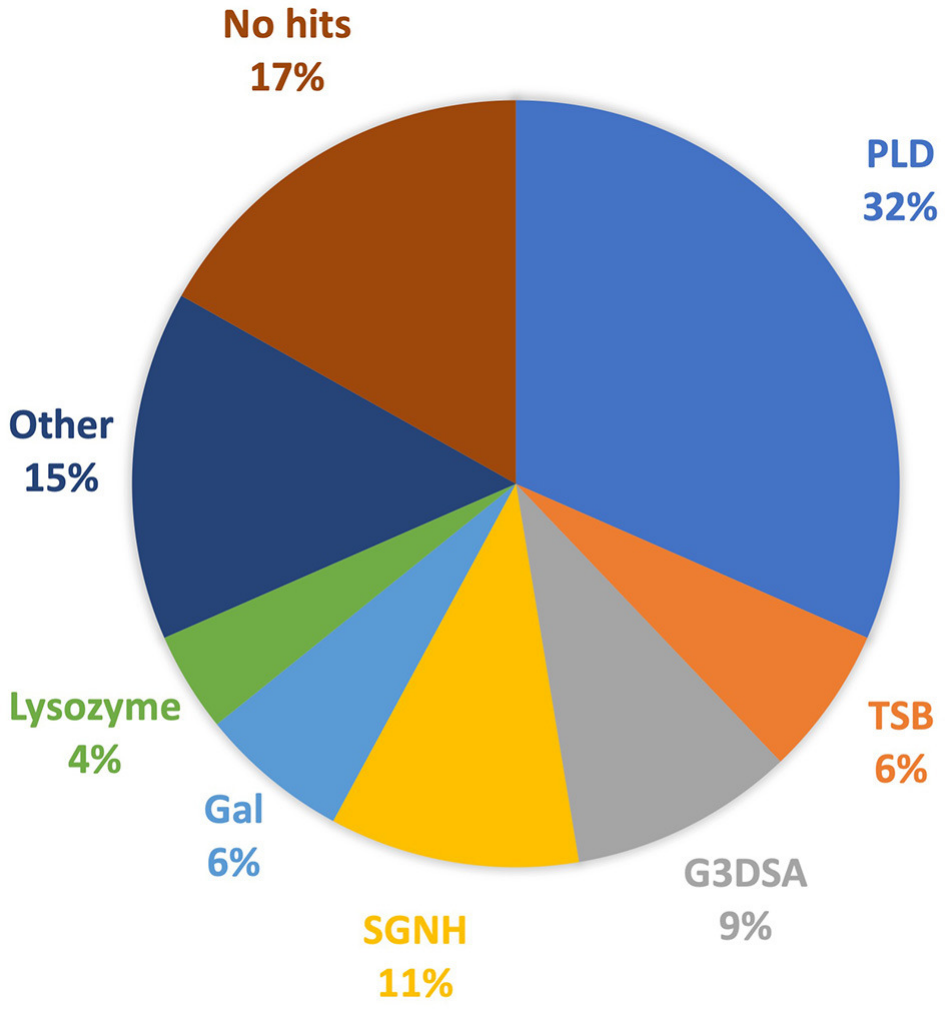
Protein domains identified in *A. baumannii* tail fibers analyzed with InterProScan: pectin lyase-like domain (PLD; blue), tailspike binding protein (TSB; orange), G3DSA:2.60.40.3940 (G3DSA; gray), SGNH domain (yellow), galactose-binding domain (Gal; light blue), lysozyme (green), other (navy), and no hits (brown).

Investigation into each functional domain identified was conducted, and information on the presence of a halo around the plaques produced by each phage, or expression of a functional depolymerase, was used. Six major functional domains were identified in 65 tail fibers: lysozyme, G3DSA:2.60.40.3940 (immunoglobulin-like), galactose-binding domain-like, pectin lyase-like (PLD), SGNH hydrolase, and phage_tailspike_middle. Less common domains, such as Concanavalin A-like lectins/glucanases, (trans)glycosidases, and peptidase_S74_CIMCD, were found in 13 tail fibers, and 16 tail fibers had no protein domain hits (Figure 1). The functional domains associated with halo formation, a common identifiable characteristic of bacteriophage depolymerases, were found to be pectin lyase-like and phage_tailspike_middle. Other domains potentially associated with depolymerase activity were (trans)glycosidases and SGNH hydrolases.

#### 3.2.1 Pectin lyase-like domains

Polysaccharide lyases, including pectin and pectate lyases, are enzymes that cleave (1,4)-glycosidic bonds through a β-elimination mechanism (Sutherland, 1999). The predominant functional domain identified in the analyzed tail fibers is the pectin lyase-like domain (PLD), which is present in 30 tail fibers. This domain is exclusive to *Ackermannviridae* (1), *Autographiviridae* (22), and myoviruses (seven) (Table 2), which are associated with depolymerase activity studied in various recombinantly expressed proteins (Liu et al., 2019b; Oliveira et al., 2019; Popova et al., 2020a; Abdelkader et al., 2022).

**Table 2:** InterProScan hit table of the 30 tail fibers containing a pectin lyase-like domain.

**Sequence**	**Name**	**Type**	**InterPro ID**	**Minimum**	**Maximum**	**Length**
AB1; gp76	Pectin_lyas_fold	Gene3D	IPR012334	155	503	349
	Beta_helix	Pfam	IPR039448	262	426	165
	pbh1	SMART	IPR006626	368	648	156
	Pectin lyase-like	SUPERFAMILY	IPR011050	201	535	335
phiAC-1; gp69	Pectin lyase-like	SUPERFAMILY	IPR011050	153	422	270
Abp1; gp47	pyocin_knob	CDD		671	761	91
	Pectin_lyas_fold	Gene3D	IPR012334	283	624	342
	Pectin lyase-like	SUPERFAMILY	IPR011050	291	615	325
IME-AB2; gp71	pyocin_knob	CDD		599	685	87
	Pectin_lyas_fold	Gene3D	IPR012334	207	547	341
	Pectin lyase-like	SUPERFAMILY	IPR011050	214	544	331
AB3; gp4	pbh1	SMART	IPR006626	420	683	92
	Pectin lyase-like	SUPERFAMILY	IPR011050	277	566	290
Petty; gp39	pyocin_knob	CDD		703	775	73
	Pectin_lyas_fold	Gene3D	IPR012334	329	586	258
	Pectin lyase-like	SUPERFAMILY	IPR011050	333	604	272
Acibel007; gp46	Pectin_lyas_fold	Gene3D	IPR012334	186	474	289
YMC-13-01-C62; gp45	Pectin lyase-like	SUPERFAMILY	IPR011050	234	545	312
YMC13/03/R2096; gp34	Pectin_lyas_fold	Gene3D	IPR012334	347	814	468
	Pectin lyase-like	SUPERFAMILY	IPR011050	376	660	285
YMC11/12/R2315; gp83	Pectin lyase-like	SUPERFAMILY	IPR011050	234	545	312
YMC11/12/R1215; gp21	Pectin lyase-like	SUPERFAMILY	IPR011050	234	545	312
Fri1; gp49	Pectin_lyas_fold	Gene3D	IPR012334	292	765	474
	Pectin lyase-like	SUPERFAMILY	IPR011050	292	623	332
phiAB6; gp40	Pectin_lyas_fold	Gene3D	IPR012334	192	387	196
	Pectate_lyase_3	Pfam	IPR024535	225	433	209
	Pectin lyase-like	SUPERFAMILY	IPR011050	221	495	275
IME200; gp48	Pectin_lyas_fold	Gene3D	IPR012334	155	493	339
	Pectin lyase-like	SUPERFAMILY	IPR011050	171	469	299
SH-Ab 15519; gp45	Pectin_lyas_fold	Gene3D	IPR012334	142	488	347
	Pectate_lyase_3	Pfam	IPR024535	176	383	208
	Pectin lyase-like	SUPERFAMILY	IPR011050	171	468	298
AS11; gp45	Pectin_lyas_fold	Gene3D	IPR012334	291	767	477
	Pectin lyase-like	SUPERFAMILY	IPR011050	292	621	330
B1; gp45	phage_tailspike_middle	CDD		173	581	409
	Pectin lyase-like	SUPERFAMILY	IPR011050	328	564	237
B3; gp42	Pectin_lyas_fold	Gene3D	IPR012334	203	561	359
	Pectate_lyase_3	Pfam	IPR024535	225	433	209
	Pectin lyase-like	SUPERFAMILY	IPR011050	222	557	336
P2; gp48	Pectin_lyas_fold	Gene3D	IPR012334	230	541	312
	Beta_helix	Pfam	IPR039448	298	462	165
	pbh1	SMART	IPR006626	377	684	180
	Pectin lyase-like	SUPERFAMILY	IPR011050	231	567	337
D2; gp2	Pectin_lyas_fold	Gene3D	IPR012334	203	569	367
	Pectate_lyase_3	Pfam	IPR024535	225	432	208
	Pectin lyase-like	SUPERFAMILY	IPR011050	221	556	336
SH-Ab 15599; gp196	Pectin_lyas_fold	Gene3D	IPR012334	302	666	365
	Pectate_lyase_3	Pfam	IPR024535	322	425	104
	Pectin lyase-like	SUPERFAMILY	IPR011050	316	656	341
Aci08; gp46	Pectin_lyas_fold	Gene3D	IPR012334	229	506	278
	pbh1	SMART	IPR006626	336	428	86
	Pectin lyase-like	SUPERFAMILY	IPR011050	328	571	244
APK2; gp43	Pectin_lyas_fold	Gene3D	IPR012334	155	493	339
	Pectin lyase-like	SUPERFAMILY	IPR011050	171	469	299
APK93; gp43	Pectin_lyas_fold	Gene3D	IPR012334	155	493	339
	Pectin lyase-like	SUPERFAMILY	IPR011050	171	469	299
APK37; gp44	Pectin_lyas_fold	Gene3D	IPR012334	225	586	362
	Pectate_lyase_3	Pfam	IPR024535	238	364	127
	Pectin lyase-like	SUPERFAMILY	IPR011050	233	542	310
PMK34; gp45	Pectin_lyas_fold	Gene3D	IPR012334	203	570	368
	Pectate_lyase_3	Pfam	IPR024535	225	433	209
	Pectin lyase-like	SUPERFAMILY	IPR011050	221	557	337
APK44; gp44	Pectin lyase-like	SUPERFAMILY	IPR011050	204	490	287
APK87; gp48	Pectin lyase-like	SUPERFAMILY	IPR011050	234	526	293
APK89; gp46	Pectin_lyas_fold	Gene3D	IPR012334	206	612	407
	Pectin lyase-like	SUPERFAMILY	IPR011050	260	566	307
Ab124; gp46	Pectin_lyas_fold	Gene3D	IPR012334	140	492	353
	Pectate_lyase_3	Pfam	IPR024535	176	383	208
	Pectin lyase-like	SUPERFAMILY	IPR011050	171	468	298

Tail fibers containing PLD exhibit diversity, with an average pairwise identity of 19.2% and lengths ranging from 693 to 921 amino acids (AAs). Among these tail fibers, 23 phages with a PLD domain were explicitly documented to exhibit halo formation around their plaques (Table 1). Although the majority of tail fibers share a common PLD SUPERFAMILY (SSF51126), phage Acibel007 (gp46) deviates with only a Gene3D hit (2.160.20.10; Pectin_lyas_fold) (Table 2). Some variations involve Pfam, SMART, or CDD hits, with 10 tail fibers overlapping a Pfam hit, eight featuring Pectate_lyase_3 (PF12708), and two labeled as Beta_helix (PF13229) for myovirus phage AB1 (gp76) and *Autographiviridae* phage P2 (gp48). Additionally, four tail fibers contain a SMART PbH1 (SM00710) hit, corresponding to parallel beta-helix repeats in pectate lyases and rhamnogalacturonase A.

**Figure 2:**
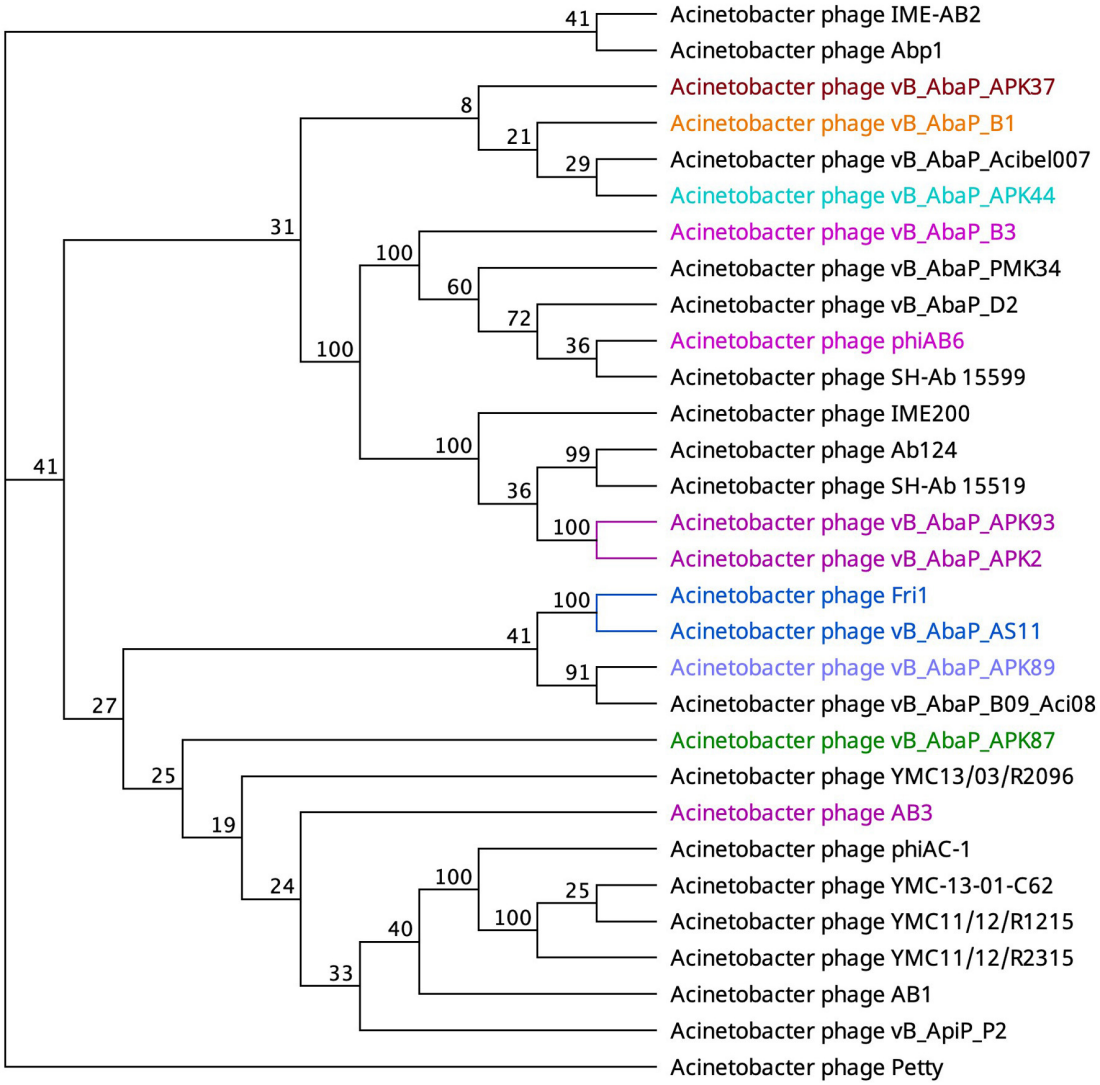
Unrooted phylogenetic tree of PLD containing tail fiber proteins with bootstrap support percent at branch nodes. The colors of tips correspond to documented capsule targets of the depolymerases: blue, K19; pink, K2; teal, K44; orange, K9; green, K87; purple, K89; and burgundy, K37.

An alternate domain architecture features a PLD SUPERFAMILY (SSF51126) overlapping a Gene3D (2.160.20.10) and a CDD pyocin_knob hit at the CTD (cd19958) (Table 2). This layout is present in the tail fibers of two podoviruses (Abp1 gp47 and IME-AB2 gp71) and one myovirus (Petty gp39) (Table 2). Phage proteins sharing this domain range from 21.9% to 29.6% identity (Supplementary Table 2). Finally, *Autographiviridae* phage B1 gp45 encoded the only tail fiber with both a PLD SUPERFAMILY hit and a phage_tailspike_middle hit from the CDD database (Table 2). A phylogenetic tree illustrates that tail fiber proteins do not group based on capsule targets but mainly on viral morphology (Figure 2). Generally, the myoviruses group together except for SH-Ab 155599 and IME-AB2, which group with podoviruses.

#### 3.2.2 Phage tailspike middle

The tail fibers of six phages feature a hit to the phage_tailspike_middle domain from the CDD (Table 3). This model characterizes the middle beta-helical domain of *Acinetobacter* bacteriophage tail spike proteins, encompassing a distinct N-terminal domain unrelated to the beta-helical substructure but implicated in virion binding. The C-terminal domain, highly variable, is suggested to play a role in receptor binding. Among these phages, AM24, IME285, BS46, and WCHABP12 (Tables 1, 3) exhibit halo formation around their plaques, with recombinantly expressed tail spike proteins from AM24 (gp50), BS46 (gp47), and IME285 (AYP68900.1) that are confirmed as functional depolymerases (Popova et al., 2019; Knirel et al., 2020; Wang et al., 2020).

**Table 3:** InterProScan hit table of the six tail fibers containing a phage_tailspike_middle domain.

**Sequence name**	**Name**	**Type**	**Min (AA)**	**Max (AA)**	**Length (AA)**
AM24; gp50	phage_tailspike_middle	CDD	266	669	404
WCHABP12; gp16	phage_tailspike_middle	CDD	150	553	404
B1; gp45	phage_tailspike_middle	CDD	173	581	409
	Pectin lyase-like	SUPERFAMILY	328	564	237
B5; gp47	phage_tailspike_middle	CDD	179	581	403
IME285; gp35	phage_tailspike_middle	CDD	150	553	404
BS46; gp47	phage_tailspike_middle	CDD	243	646	404

**Figure 3:**
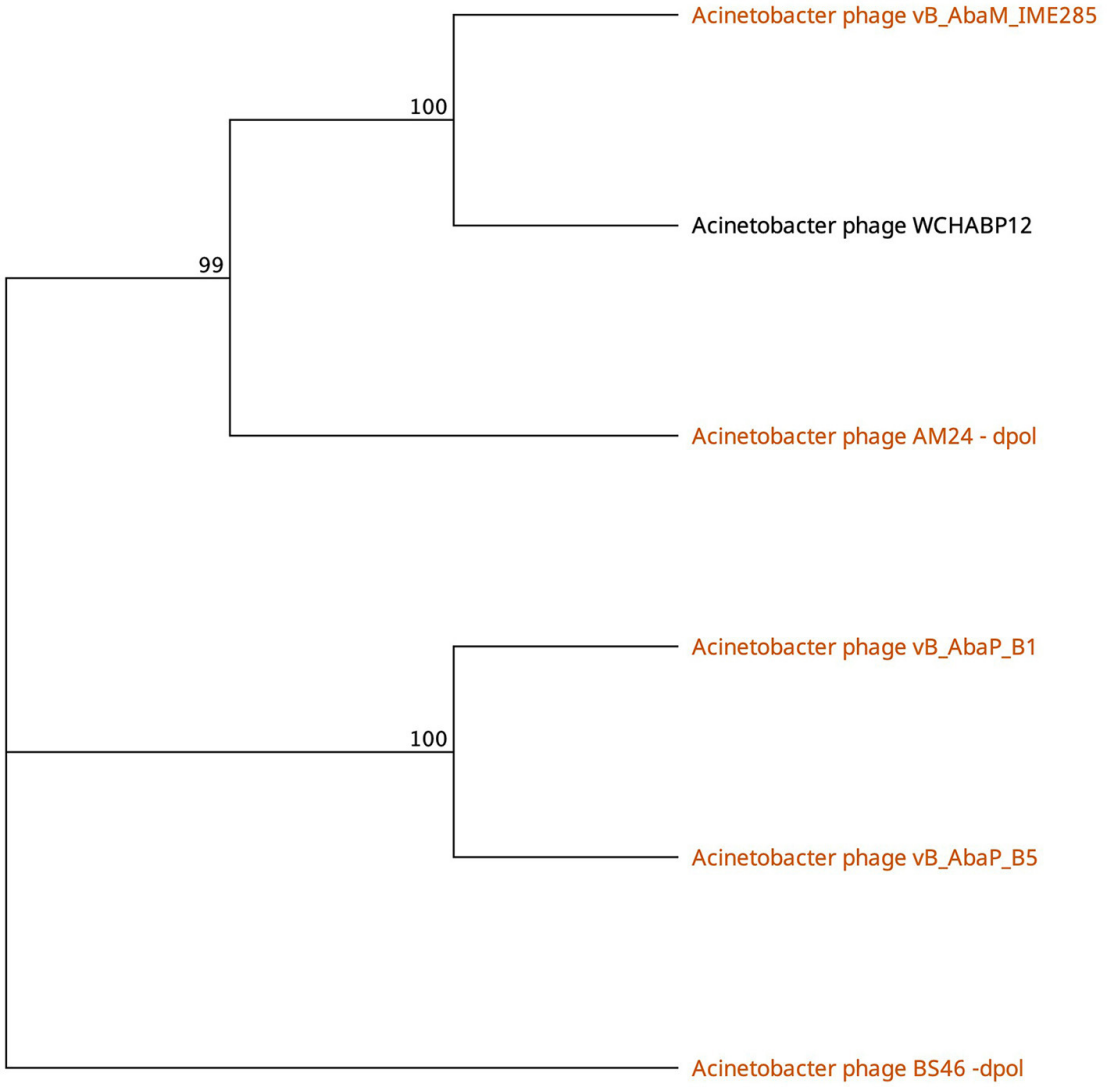
Unrooted phylogenetic tree of Tailspike_middle_domain containing tail fiber proteins with bootstrap support percent at branch nodes. Orange tips indicate halo formation documented against K9 capsule types.

Phages featuring tail fibers with the phage_tailspike_middle domain are confined to myoviruses and *Autographiviridae* lineages. The six identified proteins, ranging from 732 to 848 amino acids, share a pairwise identity of 60.1% (Supplementary Table 3). These proteins cluster by taxonomy, with a high % identity observed within myovirus-derived tail fibers (WCHABP12, gp16; IME285, AYP68900.1; BS46, gp47; and AM24, gp50) at 72.4–97.4% (Figure 3, Supplementary Table 2). *Autographiviridae* proteins B1 (gp45) and B5 (gp47) share 40.7% identity (Figure 3, Supplementary Table 3). Intriguingly, B5′s tail fiber exhibits greater similarity to myovirus phage tail fibers, with % identity ranging from 69.1% to 73.2%. The most diverse tail fiber in this group belongs to B1, featuring a SUPERFAMILY pectin lyase-like hit (SSF51126) overlapping the CDD domain (Figure 3, Table 3). This 761-AA-long tail fiber shares 22.5–40.7% identity with other tail fibers in this group.

#### 3.2.3 CATH/G3DSA 2.60.40.3940 domain

The CATH superfamily 2.60.40.3940 is characterized by a predominantly beta class (2), sandwich architecture (2.60), and immunoglobulin-like topology (2.60.40) within a novel homologous superfamily (2.60.40.3940). In the *Obolenskvirus* clade of *Acinetobacter* myophages, nine phages, namely, AP22 (gp53), AB1 (gp77), YMC-13-01-C62 (gp46), YMC11/12/R2315 (gp84), YMC11/12/R1215 (gp20), WCHABP12 (gp15), WCHABP1 (gp6), AbP2 (gp18), and Abp9 (gp49), feature the G3DSA 2.60.40.3940 domain in the CTD of one of their tail fiber proteins (Table 4). These proteins, encoded in the morphogenesis region and positioned upstream of other tail fiber proteins, vary from 258 to 283 amino acids, sharing an overall pairwise identity of 83.0% (Supplementary Table 4). An analysis of the multisequence alignment reveals high sequence conservation at the N-terminal domain (NTD), indicating its involvement in virion attachment. Conversely, the CTD, associated with the 2.60.40.3940 hit, displays a breakdown in sequence identity among some proteins, suggesting a potential role in host cell recognition. This role is supported by the crystal structure of *Acinetobacter* phage AP22 gp53 CTD (PDB: 4MTM), resembling a homotrimeric globular lectin-like protein with a slender midsection, akin to host-cell binding proteins of *Escherichia coli* phages T7 gp17 (Garcia-Doval and Van Raaij, 2012) and T4 gp12 (Van Raaij et al., 2001). Furthermore, AP22 gp53C binds to ethylene glycol and glycerol molecules, which are surrogates of an oligosaccharide backbone. Overall, this data suggests that tail fibers encoding a 2.60.40.3940 domain are involved in recognizing oligosaccharide moieties of *Acinetobacter baumannii*.

**Table 4:** InterProScan hit table of the nine tail fibers containing a CATH/G3DSA 2.60.40.3940 domain.

**Sequence Name**	**Name**	**Type**	**Min (AA)**	**Max (AA)**	**Length (AA)**
AP22; gp53	G3DSA:2.60.40.3940	Gene3D	176	271	96
AB1; gp77	G3DSA:2.60.40.3940	Gene3D	184	283	100
	Agglutinin HPA-like	SUPERFAMILY	192	282	91
C62; gp46	G3DSA:2.60.40.3940	Gene3D	181	276	96
R2315; gp84	G3DSA:2.60.40.3940	Gene3D	182	276	95
R1215; gp20	G3DSA:2.60.40.3940	Gene3D	181	276	96
WCHABP12; gp15	G3DSA:2.60.40.3940	Gene3D	187	281	95
WCHABP1; gp6	G3DSA:2.60.40.3940	Gene3D	187	281	95
AbP2; gp18	G3DSA:2.60.40.3940	Gene3D	183	276	94
Abp9; gp49	G3DSA:2.60.40.3940	Gene3D	165	258	94

#### 3.2.4 SGNH hydrolase domain

**Figure 4:**
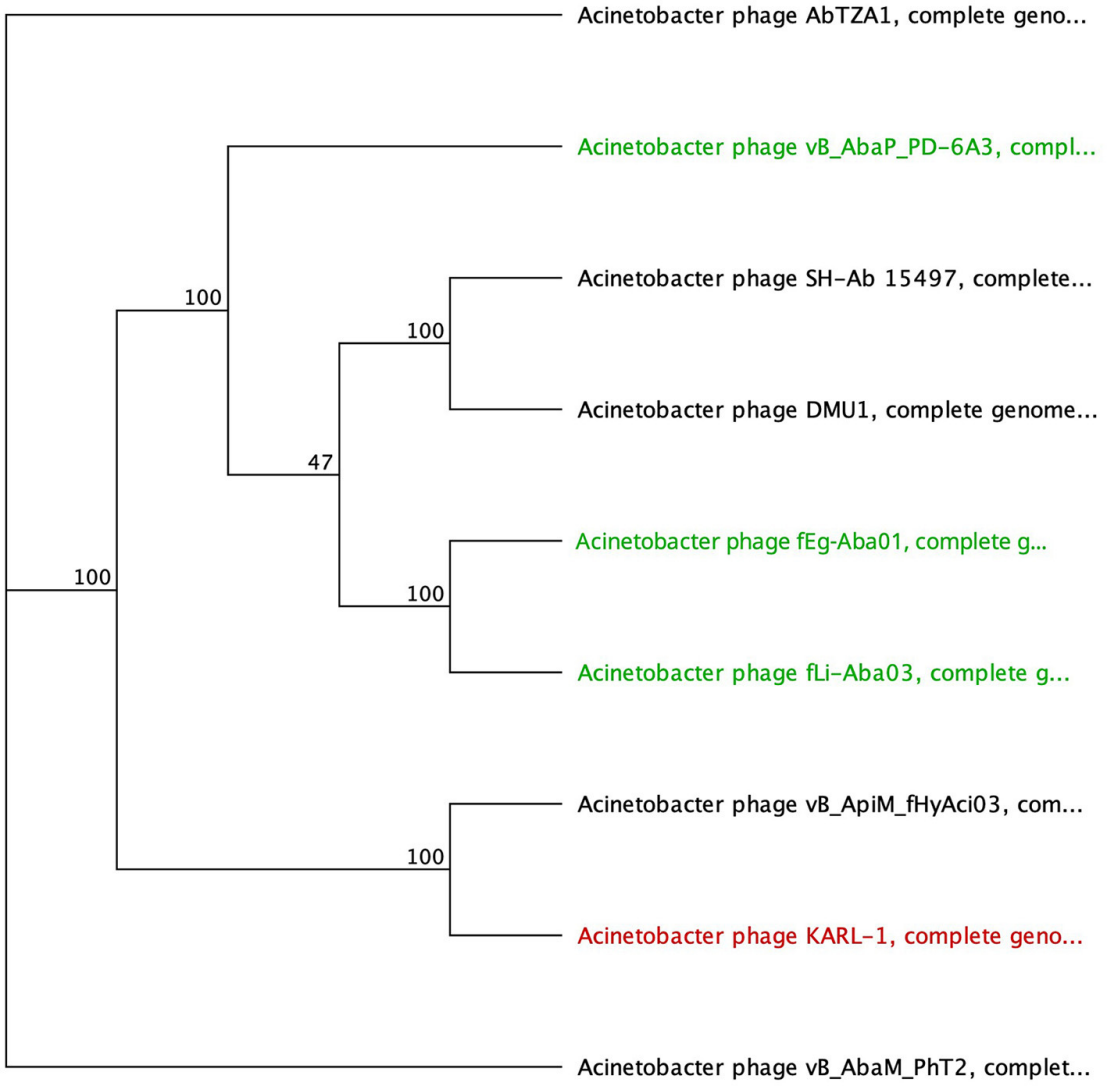
Unrooted phylogenetic tree of G3DSA:2.60.40.3940 domain containing tail fiber proteins with bootstrap support percent at branch nodes. Green tips indicate halo formation documented in the literature.

The SGNH hydrolase superfamily comprises 16 well-studied protein families with a conserved catalytic fold and mechanism (Anderson et al., 2022). These enzymes, named after their catalytic Ser, His, Gly, and Asn residues, function as esterases and lipases, playing vital roles in biomass conversion, pathogenesis, and cell signaling (Akoh et al., 2004; Anderson et al., 2022). The SGNH hydrolase domain was identified in the tail fibers of 10 phages: SH-Ab 15497, fHyAci03, KARL-1, PhT2, fEg-Aba01, fLi-Aba02, fLi-Aba03, DMU1, PD-6A3, and AbTZA1, with a pairwise identity of 33.7%. Four of these phages exhibited halo formation (PD-6A3, fEg-Aba01, fLi-Aba02, and fLi-Aba03) (Wu et al., 2019; Badawy et al., 2020) (Table 5, Supplementary Table 5). Based on the structural organization of the domains, the tail fibers can be grouped into two architectures. One group features two fibritin domains: one at the NTD and one directly upstream of the SGNH hydrolase domain (Table 5). This domain layout is restricted to four members of the subfamily *Tevenvirinae:* vB_ApiM_fHyAci03 (fHyAci03), KARL-1, vB_AbaM_PhT2 (PhT2), and AbTZA1, which tend to group together (Figure 4). Fibritin belongs to a class of chaperones that catalyze specific phage-assembly processes, promoting the assembly of the long tail fibers and their attachment to the tail baseplate (Tao et al., 1997). Furthermore, fibritin also serves as a sensing device, controlling the retraction of the long tail fibers in adverse environments to prevent infection (Tao et al., 1997). These four proteins with this domain layout have similar lengths, ranging from 621 to 626 AA and share between 55% and 97.6% AA identity (Table 5 and Supplementary Table 5).

**Table 5:** InterProScan hit table of the 10 tail fibers containing an SGNH hydrolase domain.

**Sequence**	**Name**	**Type**	**Min (AA)**	**Max (AA)**	**Length (AA)**
SH-Ab 15497; gp21	SGNH hydrolase	Gene3D	267	522	256
	SASA	Pfam	358	519	162
	SGNH hydrolase	SUPERFAMILY	338	520	183
fHyAci03; gp175	SGNH hydrolase	Gene3D	463	615	153
	G3DSA:1.20.5.320	Gene3D	353	446	94
	G3DSA:1.20.5.320	Gene3D	3	89	87
	Fibritin_C	Pfam	357	412	56
	SGNH hydrolase	SUPERFAMILY	472	606	135
	Fibritin	SUPERFAMILY	6	106	101
KARL-1; gp114	SGNH hydrolase	Gene3D	466	618	153
	G3DSA:1.20.5.320	Gene3D	356	449	94
	G3DSA:1.20.5.320	Gene3D	3	89	87
	Fibritin_C	Pfam	360	415	56
	SGNH hydrolase	SUPERFAMILY	475	609	135
	Fibritin	SUPERFAMILY	6	106	101
PhT2; gp163	SGNH hydrolase	Gene3D	450	624	175
	G3DSA:1.20.5.320	Gene3D	357	449	93
	G3DSA:1.20.5.320	Gene3D	2	89	88
	Fibritin_C	Pfam	362	416	55
	SGNH hydrolase	SUPERFAMILY	476	601	126
	Fibritin	SUPERFAMILY	4	106	103
fEg-Aba01; gp19	SGNH hydrolase	Gene3D	258	481	224
	SASA	Pfam	315	477	163
	SGNH hydrolase	SUPERFAMILY	233	479	247
fLi-Aba02; gp21	SGNH hydrolase	Gene3D	258	481	224
	SASA	Pfam	315	477	163
	SGNH hydrolase	SUPERFAMILY	233	479	247
fLi-Aba03; gp21	SGNH hydrolase	Gene3D	258	481	224
	SASA	Pfam	315	477	163
	SGNH hydrolase	SUPERFAMILY	233	479	247
DMU1; gp20	SGNH hydrolase	Gene3D	267	521	255
	SASA	Pfam	356	518	163
	SGNH hydrolase	SUPERFAMILY	337	519	183
PD-6A3; gp13	SGNH hydrolase	Gene3D	430	682	253
	SASA	Pfam	522	664	143
	SGNH hydrolase	SUPERFAMILY	427	675	249
AbTZA1; gp180	SGNH hydrolase	Gene3D	450	624	175
	G3DSA:1.20.5.320	Gene3D	357	449	93
	G3DSA:1.20.5.320	Gene3D	2	89	88
	Fibritin_C	Pfam	361	416	56
	SGNH hydrolase	SUPERFAMILY	476	601	126
	Fibritin	SUPERFAMILY	4	106	103

The second group comprises hypothetical proteins from six phages, featuring a lone SGNH domain at the CTD of the protein (Figure 4, Table 5). This domain structure is present in one *Autographiviridae* member, PD-6A3, and five siphoviruses (SH-Ab 15497, fEg-Aba01, fLi-Aba02, fLi-Aba03, DMU1), which group more closely together (Figure 4). PD-6A3 encodes an endolysin with activity against *A. baumannii* cells, which could potentially be responsible for the observed halo formation (Wu et al., 2019). In contrast, siphophages, fEg-Aba01, fLi-Aba02, and fLi-Aba03, encode two putative tail fiber proteins: one with an SGNH hydrolase domain and, in the case of fLi-Aba02 and fLi-Aba03, a Concanavalin A-like lectins/glucanases domain. The siphovirus proteins are very close in length, ranging from 620 to 660 AA, sharing 41 to 100% AA identity (Table 5, Supplementary Table 5). Phages with 100% AA identity are fEg-Aba01, fLi-Aba02, and fLi-Aba03. Comparatively, the podophage PD-6A3 tail fiber protein is significantly longer at 817 AA, although it shares approximately 41% identity with the five siphoviruses of the same layout (Figure 4, Table 5, Supplementary Table 5).

Since our data collection, the *Acinetobacter* podovirus Aristophanes was published. This phage does not produce a halo but encodes a tail spike SGNH hydrolase domain (gp41) (Timoshina et al., 2021). Functional study of this protein revealed a tail deacetylase causing O-acetylation of one of the K26 sugar residues which causes a slight decrease in turbidity of the host (Timoshina et al., 2021). This finding suggests that the other phages may also utilize this structural protein as a deacetylase. To get more information on the potential functions of the phages DMU1, PD-6A3, and SH Ab 15497, the proteins were modeled with AlphaFold Multimer as homotrimers, and the resulting models were submitted to the DALI server PDB search. The top hits are to a xyloglucan-active beta-galactosidase from *Xanthomonas citri* (7KMM) for all models, followed by a protein with an unknown function from *Arabidopsis thaliana* (2APJ) for DMU1 and PD-63A and a homo-dimer acetylxylan esterase from *Clostridium acetobutylicum* (1ZMB) for SH Ab 15497.

#### 3.2.5 Galactose binding domain

**Figure 5:**
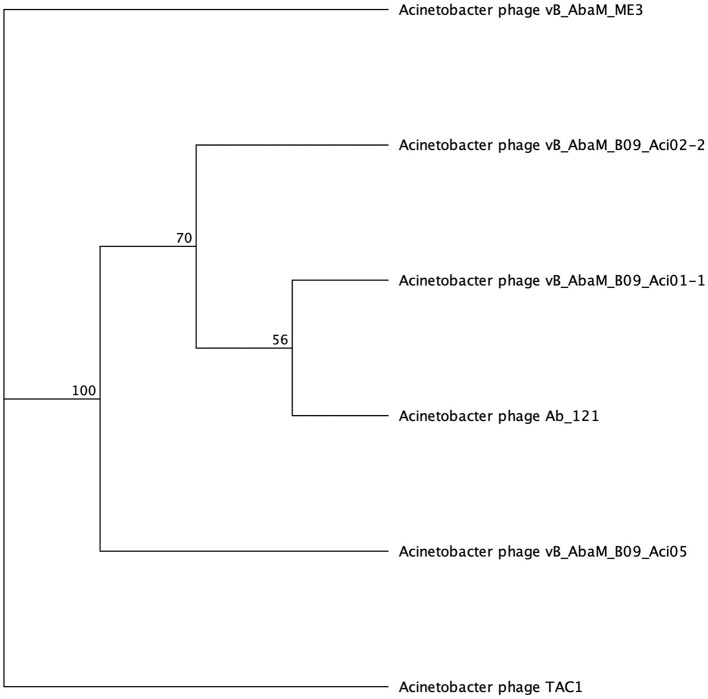
Unrooted phylogenetic tree of SGNH hydrolase-encoding tail fiber proteins. Bootstrap support percent is listed at branch nodes. Green tips indicate halo formation in literature, red tips indicate lack of halo formation documented, and black tips indicate no data on halo formation available.

Galactose binding domains are present in several different protein families in eukaryotes and prokaryotes and bind to specific ligands, such as cell-surface-attached carbohydrates (Ito et al., 1991). The members of this domain exhibit a β-sandwich forming a jelly roll fold. In the tail fibers of six myophages (ME3, TAC1, Aci05, Aci02-2, Aci01-1, and Ab_121), a galactose-binding domain was identified (Figure 5, Table 6). Notably, literature reports indicate that five of these phages do not exhibit halo formation (Lee et al., 2011; Essoh et al., 2019; Asif et al., 2020). Tail fibers encoding this domain varied significantly in sequence length, spanning from 1,177 to 5,419 AA (Table 6), with diverse percent amino acid identity ranging from 12.9 to 98.8% (Supplementary Table 6). ME3, the first jumbo *Acinetobacter* phage identified, presented the most divergent tail fiber with an average % identity ranging from 12.4–13.2 % (Figure 5, Supplementary Table 6) (Buttimer et al., 2016). The tail fiber protein encoded by ME3 is 5,419 AA long and features multiple hits from Gene3D, SUPERFAMILY, Pfam, and PROSITE profiles (Figure 5, Table 5).

**Table 6:** InterProScan hit table of the six tail fibers containing a galactose binding domain.

**Sequence name**	**Name**	**Type**	**Min (AA)**	**Max (AA)**	**Length (AA)**
ME3; gp104	G3DSA:2.60.120.260	Gene3D	4,468	4,628	161
	G3DSA:2.60.120.260	Gene3D	1,014	1,172	159
	G3DSA:2.60.120.260	Gene3D	4,179	4,331	153
	G3DSA:2.60.120.260	Gene3D	3,438	3,577	140
	G3DSA:2.60.120.260	Gene3D	5,266	5,398	133
	G3DSA:2.60.120.260	Gene3D	5,018	5,128	111
	F5_F8_type_C	Pfam	4,505	4,608	104
	DUF1983	Pfam	4,359	4,410	52
	FA58C_3	PROSITE_PROFILES	4,461	4,623	163
	Galactose-binding domain-like	SUPERFAMILY	4,178	4,331	154
	Galactose-binding domain-like	SUPERFAMILY	4,483	4,622	140
	Galactose-binding domain-like	SUPERFAMILY	5,264	5,397	134
	Galactose-binding domain-like	SUPERFAMILY	3,442	3,571	130
Aci05; gp30	G3DSA:2.60.120.260	Gene3D	800	922	123
	Galactose-binding domain-like	SUPERFAMILY	798	895	98
Aci01-1; gp31	G3DSA:2.60.120.260	Gene3D	1,265	1,398	134
	G3DSA:2.60.120.260	Gene3D	800	922	123
	A-type inclusion protein, putative-related	Panther	424	1,488	1,065
	Galactose-binding domain-like	SUPERFAMILY	800	878	79
Aci02-2; gp32	G3DSA:2.60.120.260	Gene3D	1,265	1,398	134
	G3DSA:2.60.120.260	Gene3D	800	922	123
	A-type inclusion protein, putative-related	Panther	423	1,485	1,063
	Galactose-binding domain-like	SUPERFAMILY	800	878	79
TAC1; gp42	G3DSA:1.20.5.340	Gene3D	920	1,470	221
	G3DSA:2.60.120.260	Gene3D	777	919	143
	G3DSA:1.10.287.1490	Gene3D	427	563	137
	Myosin heavy chain - related	Panther	184	1,020	837
	MPN010-like	SUPERFAMILY	1,408	1,460	53
Ab 121; gp12	G3DSA:2.60.120.260	Gene3D	1,265	1,398	134
	G3DSA:2.60.120.260	Gene3D	800	922	123
	A-type inclusion protein, putative-related	Panther	91	1,436	1,346
	Galactose-binding domain-like	SUPERFAMILY	798	895	98

The second most divergent tail fiber belongs to TAC1, displaying an identity range of 13–42.5% (Figure 5, Supplementary Table 6), containing a myosin heavy chain Panther hit (PTHR18921) overlapping a Gene3D hit (1.10.287.1490, Phosphatidylinositol 3-kinase regulator activity). TAC1′s tail fiber also included two additional Gene3D hits corresponding to a galactose-binding domain (2.60.120.260) and a myosin heavy chain (1.20.5.340). Phages Ab_121, Aci01-1, and Aci02-2 encode a 2,211 AA tail fiber protein with an identity range of 96.8% and 98.9% (Supplementary Table 6). Aci01-1 and Aci02-2 have matching Panther (A-type inclusion protein), Gene3D (galactose binding domain), and SUPERFAMILY (galactose binding domain) hits spanning the length of the protein (Figure 5, Table 6). Phage Ab_121 tail fiber has the same Gene3D hits but differs in the location of the Panther and SUPERFAMILY hits. Phage Aci05 also has similar domain hits to phages Ab_121, Aci01-1, and Aci02-2, although it lacks a Panther and one of the Gene3D hits.

#### 3.2.6 Lysozyme domain

Four phages were identified to encode a lysozyme domain in their tail fibers (Table 6). Among them, three are unclassified *Tevenvirinae* myoviruses (ZZ1, gp162; fHyAci03, gp165; and KARL-1, gp124) while the remaining phage is an unclassified podovirus (Presley, gp80). Notably, only the characterization article of KARL-1 discussed halo formation, which was not observed previously (Jansen et al., 2018). Presley shares 12% identity to the myovirus proteins (Supplementary Table 7).

**Figure 6:**
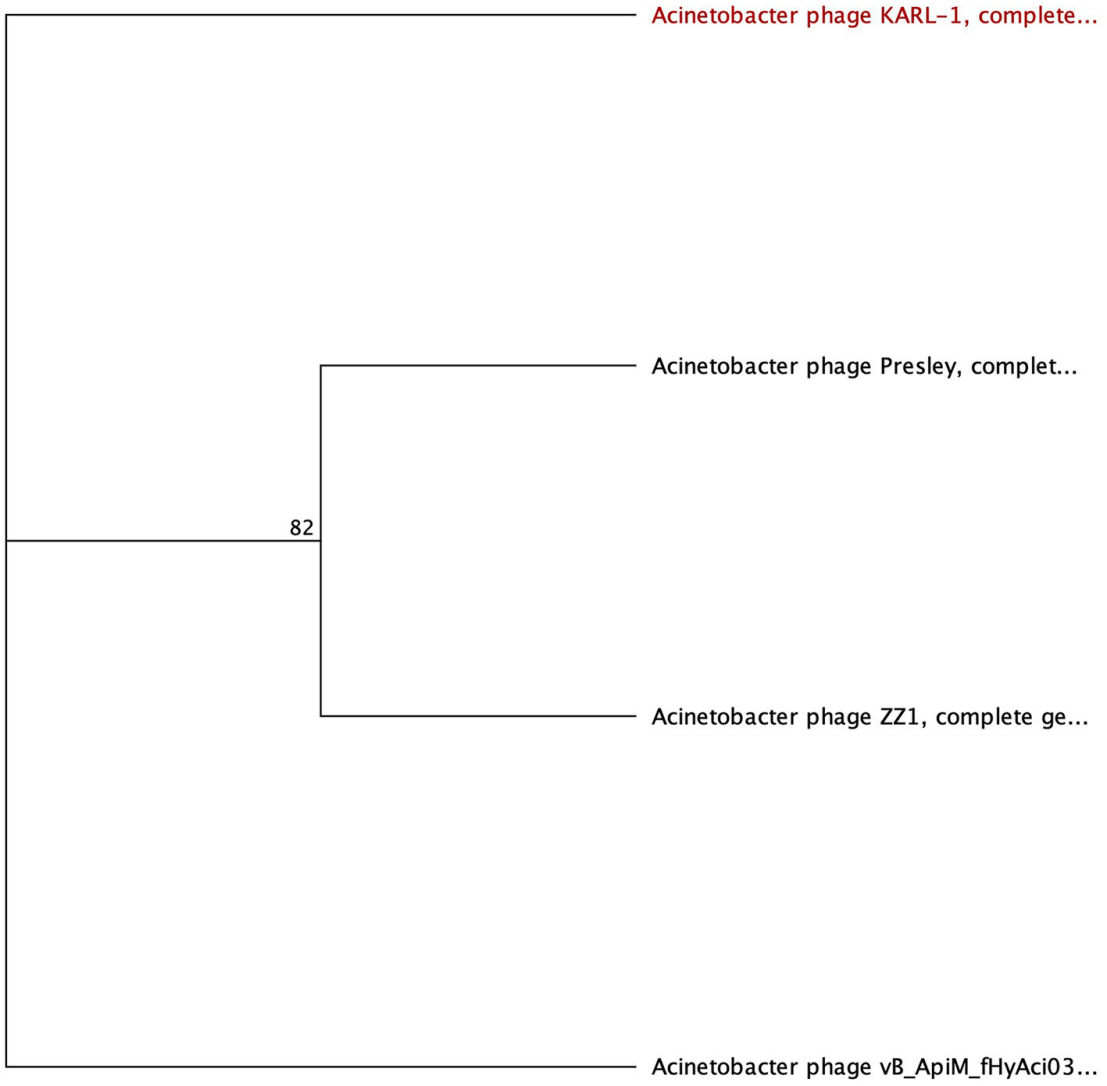
Unrooted phylogenetic tree of lysozyme-encoding tail fiber proteins. Bootstrap support percent is listed at branch nodes. Red tips indicate a lack of halo formation documented, and black tips indicate no data on halo formation available.

The most highly annotated proteins with the lysozyme domain belong to *Tevenvirinae*, reflecting the extensive research on the classical coliphage T4 (Table 7). The sequence length of these proteins varies from 593 to 600 AA long with notable conservation in percent identity ranging from 83.4% and 99.7% (Supplementary Table 7). All three proteins hit needle_T4 from the HAMAP database (Figure 6). Furthermore, each phage has three distinct SUPERFAMILY database hits corresponding to gp5 N-terminal domain-like (SSF69255), lysozyme-like (SSF53955), and phage fiber proteins (SSF69349) (Figure 6, Table 7). Three Gene3D hits are present on all proteins (2.40.50.260, 1.10.530.40, and 3.10.450.190), as well as two Pfam hits (Gp5_OB, PF06714 and Phage_lysozyme, PF00959). The lysozyme functionality of these proteins is supported by a Panther hit (T4-type lysozyme 1-related, PTHR37406), CDD hit (T4-like_lys, cd00735), and a PRINTS hit (T4 lysozyme, PR00684).

**Table 7:** InterProScan hit table of the four tail fibers containing a lysozyme domain.

**Sequence**	**Name**	**Type**	**Min (AA)**	**Max (AA)**	**Length (AA)**
ZZ1; gp162	T4-like_lys	CDD	173	331	159
	Needle_t4	HAMAP	3	593	591
	T4lysozyme	PRINTS	175	332	121
	T4-type lysozyme 1-related	Panther	173	332	160
	Gp5_OB	Pfam	33	171	139
	Phage_lysozyme	Pfam	195	320	126
	G3dsa:1.10.530.40	Gene3D	172	337	166
	G3dsa:2.40.50.260	Gene3D	1	128	128
	G3dsa:3.10.450.190	Gene3D	390	461	72
	Phage fiber proteins	SUPERFAMILY	391	585	195
	Lysozyme-like	SUPERFAMILY	145	337	193
	Gp5 N-terminal domain-like	SUPERFAMILY	6	128	123
Presley; gp80	LT-like	CDD	256	376	121
	G3dsa:1.10.530.10	Gene3D	242	387	146
	Slt	Pfam	273	353	81
	Lysozyme-like	SUPERFAMILY	256	373	118
fHyAci03; gp165	T4-like_lys	CDD	173	331	159
	Baseplate hub and tail lysozyme CDS	CDS	1	594	594
	Needle_t4	HAMAP	3	594	592
	T4lysozyme	PRINTS	175	332	121
	T4-type lysozyme 1-related	Panther	173	332	160
	Gp5_OB	Pfam	33	171	139
	Phage_lysozyme	Pfam	195	320	126
	G3dsa:1.10.530.40	Gene3D	172	337	166
	G3dsa:2.40.50.260	Gene3D	1	128	128
	G3dsa:3.10.450.190	Gene3D	376	463	88
	Lysozyme-like	SUPERFAMILY	141	338	198
	Phage fiber proteins	SUPERFAMILY	393	586	194
	Gp5 N-terminal domain-like	SUPERFAMILY	6	128	123
KARL-1; gp124	T4-like_lys	CDD	173	331	159
	Needle_t4	HAMAP	3	594	592
	T4lysozyme	PRINTS	175	332	121
	T4-type lysozyme 1-related	Panther	173	332	160
	Gp5_OB	Pfam	33	171	139
	Phage_lysozyme	Pfam	195	320	126
	G3DSA:1.10.530.40	Gene3D	172	337	166
	G3DSA:2.40.50.260	Gene3D	1	128	128
	G3DSA:3.10.450.190	Gene3D	376	463	88
	Lysozyme-like	SUPERFAMILY	141	338	198
	Phage fiber proteins	SUPERFAMILY	393	586	194
	gp5 N-terminal domain-like	SUPERFAMILY	6	128	123

#### 3.2.7 Other domains

The analysis of the phage tail fibers also revealed nine unique functional domains found in three *Autographiviridae*, four myoviruses, and five siphovirus phages; of which, seven were reported to have halo formation: fEgAba01 (gp20), fLiAba02 (gp22), fLiAba03 (gp22), Aci07 (gp45), TaPaz (gp78), APK116 (gp43), and AS12 (gp42) (Table 8) (Popova et al., 2017, 2020a; Essoh et al., 2019; Badawy et al., 2020; Shchurova et al., 2021). Further analysis will be focused on the abovementioned phages due to their halo formation and in the case of AKP116, AS12, and TaPaz, the expression of a recombinant depolymerase enzyme. The six remaining phage tail fibers with unique domains belong to Acibel004 (gp123; 2201 AA), ME3 (gp106; 574 AA), TRS1 (gp30; 526 AA), fHyAci03 (gp247; 1259 AA), R2096 (gp66; 414 AA), and ZZ1 (gp171; 510 AA) (Table 8).

**Table 8:** InterProScan hit table of the 11 tail fibers encoding rare domains.

**Sequence name**	**Name**	**Type**	**Min (AA)**	**Max (AA)**	**Length (AA)**
YMC13/03/R2096; gp66	Collagen	Pfam	50	96	47
ME3; gp106	G3DSA:3.90.1340.10	Gene3D	418	482	65
	The receptor-binding domain of short tail fiber protein gp12	SUPERFAMILY	423	574	152
	Collar	Pfam	430	488	59
	Phage_fiber_2	Pfam	244	280	37
TRS1; gp30	G3DSA:2.10.10.20	Gene3D	75	119	45
AS12; gp42	Peptidase_S74_CIMCD	CDD	788	896	109
	G3DSA:1.10.10.10	Gene3D	788	901	114
	ICA	PROSITE_PROFILES	788	901	114
Loki; gp20	G3DSA:2.60.40.10	Gene3D	475	577	103
	FN3	PROSITE_PROFILES	481	578	98
	Phage-tail_3	Pfam	265	411	147
	Fibronectin type III	SUPERFAMILY	483	653	171
fHyAci03; gp247	pyocin_knob	CDD	334	408	75
	Phage_T4_gp36	Pfam	828	933	106
Aci07; gp45	(trans)glycosidases	SUPERFAMILY	292	498	207
APK116; gp43	pyocin_knob	CDD	645	730	86
TaPaz; gp78	Peptidase_S74_CIMCD	CDD	749	875	127
	ICA	PROSITE_PROFILES	749	878	130
	Peptidase_S74	Pfam	749	807	59
fLiAba02; gp22	G3DSA:2.60.120.200	Gene3D	82	256	175
	Concanavalin A-like lectins/glucanases	SUPERFAMILY	80	246	167
fLiAba03; gp22	G3DSA:2.60.120.200	Gene3D	82	256	175
	Concanavalin A-like lectins/glucanases	SUPERFAMILY	80	246	167

The tail fiber proteins from the podovirus AS12 (gp42, 901 AA) and the myovirus TaPaz (gp78, 878 AA) have peptidase domain hits at their CTD (Table 8). The functional domains of these tail fibers are of special interest because they have already been characterized as depolymerases (Popova et al., 2017; Shchurova et al., 2021). A PROSITE profiles hit (PS51688) was identified, which is an intramolecular chaperone auto-processing (ICA) domain that can catalyze the trimerization-dependent auto-proteolysis using two conserved serine and lysine residues. This domain has been identified in bacteriophage-encoded endosialidases and tail spike and fiber proteins (Schwarzer et al., 2007). The protein domain responsible for endosialidase activity in the ICA domain-containing tail fibers is restricted to the NTD of the proteins (Schwarzer et al., 2007). The final shared database hit overlaps the PROSITE profiles hit and is from the CDD database (cd10144) to the Peptidase S74 protein family of known phage endosialidases (Table 8). TaPaz gp78 has a Pfam Peptidase S74 hit (PF13884), while AS12 differs with an overlapping Gene3D hit (1.10.10.10), which is found with winged helix DNA-binding proteins (Table 8).

**Figure 7:**
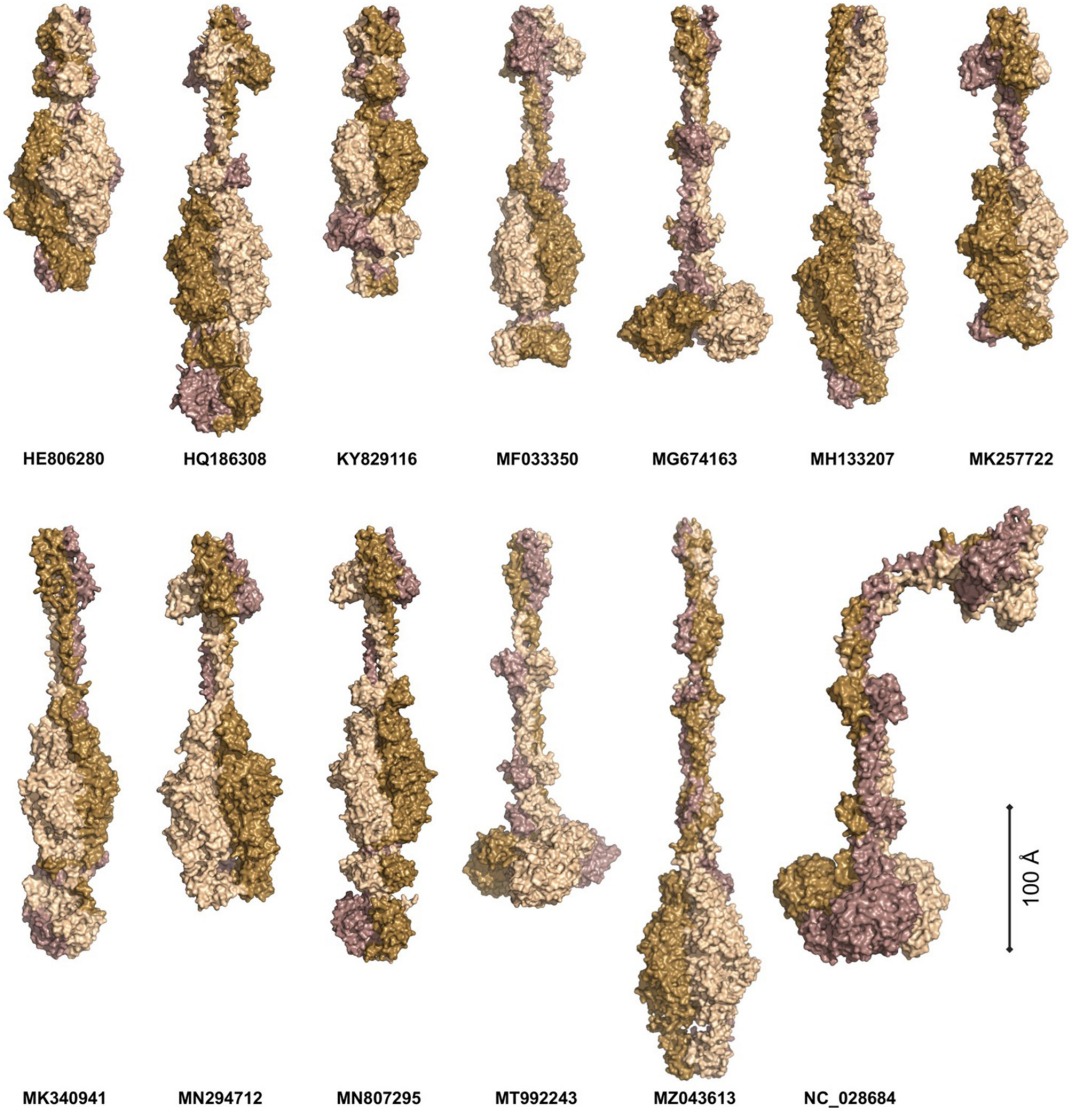
The models of *Acinetobacter* phage tail fiber proteins. The proteins were predicted as homotrimers with AlphaFold. The models were predicted with at least 90% confidence (AP22, HE806280, WCHABP1: KY829116), 80% (PhiAB1: HQ186308, P1: MF033350, B9: MH133207, APK32: MK257722, AbTj: MK340941, APK48: MN294712), 70% (SH-Ab 15497: MG674163, APK116: MN807295, DMU1: MT992243, TaPaz: MZ043613), and 60% (PD-6A3: NC_028684). The monomers are color-coded to highlight the symmetric nature of the complexes. The proteins are scaled proportionally to give an order of magnitude in size.

Phages APK116 (gp43, 861 AA) and PhiAB1 (gp41, 882 AA) both contain a lone pyocin knob domain at the CTD from CDD (cd19958) (Table 8). This domain layout is similar to those of *Friunavirus* members discussed above, except it lacks a pectin-lyase domain hit. Phage APK116 was documented to have halo formation, and gp43 was recombinantly expressed and shown to function as a depolymerase (Popova et al., 2020a). APK116 gp43 and PhiAB1 gp41 were modeled with AlphaFold and the models were submitted to the DALI server (Figure 7). The top DALI models for these two proteins are *Acinetobacter* phage AS12 depolymerase gp42 (6EU4) (Table 8), followed by the *E. coli* CAB120 depolymerase tail spike protein (6W4Q) for phiAB1 and poly(beta-d-mannuronate) c5 epimerase from *Azotobacter vinelandii* (5LW3) for APK116.

Two siphophages, fLiAba02 and fLiAba03 (gp22; identical proteins), were found to be the only phage tail fibers to have a Concanavalin A-like lectins/glucanases (CALG) SUPERFAMILY (SSF49899) domain hit (Table 8). Lectins are described as non-immune origin proteins possessing binding affinity toward glycoconjugates in a specific and reversible manner. As discussed above, these phages have an additional tail fiber encoding an SGNH hydrolase. To further investigate the function of the CALG domains, other characterized depolymerases were searched. *Paenibacillus* sp. 32352 is a soil-dwelling bacterium that produces the enzyme Pn3Pase, which degrades the capsular polysaccharide of *Streptococcus pneumoniae* serotype 3 (Pn3P) (Middleton et al., 2018; Wantuch et al., 2021). This protein encodes two SUPERFAMILY domain hits of interest: CALG (SSF49899; CTD) and (trans)glycosidases (SSF51445; NTD). Transglycosylases are a class of glycosyl hydrolase enzymes that can catalyze the transformation of one glycoside to another (Romero-Téllez et al., 2019). The functional domain responsible for the depolymerization of Pn3P was found to be the (trans)glycosidase domain, as knockouts of this region result in the loss of depolymerase activity compared to the loss of the CALG domain (Wantuch et al., 2021). The findings of the Pn3Pase mutation experiment can further be applied to phage Aci07 (gp45). As mentioned above, this phage has also documented halo formation. This is the only tail fiber with a hit to (trans)glycosidases from the SUPERFAMILY database (SSF51445) (Table 8). This is the same SUPERFAMILY domain as the Pn3Pase depolymerase discussed above, which suggests that gp45 of Aci07 may be a functional depolymerase.

#### 3.2.8 Tail fibers without domain hits

Four phage tail fibers with no domain hits or information on halo formation will not be investigated further: ABP2 gp17, TRS1 gp29, Acibel004 gp148 and IME-AB2 gp72, and PD-AB9. Six phage tail fibers had no domain hits present, although their function as depolymerases was experimentally confirmed by researchers studying their recombinant proteins. These phages include AP22 (gp54), APK32 (gp46), APK48 (gp43), P1 (gp43), B9 (gp69), and TaPaz (gp79) (Oliveira et al., 2017, 2018; Knirel et al., 2020; Popova et al., 2020a; Shchurova et al., 2021). Furthermore, halo formation was detailed in the following phages but the protein responsible was not confirmed: AbTj (gp53) and WCHABP1 (gp5) (Zhou et al., 2018; Xu et al., 2020; Drobiazko et al., 2022). All the eight abovementioned proteins were modeled as homotrimers with AlphaFold Multimer and showed narrow midsections and larger globular regions (Figure 7).

The protein models were submitted to DALI PDB search to identify similar structural models (Figure 7, Supplementary Table 8) (Jumper et al., 2021; Holm, 2022). The top P1 DALI hit is to its own crystal structure (6E1R), followed by the putative pectin lyase gp18 (7CHU) of the *Geobacillus* virus E2 (Table 8). APK32 top DALI model is to *Acinetobacter* phage AS12 gp42 (6EU4), the characterized depolymerase discussed in the above section documenting rare domains (Table 8). The AP22 DALI hit is to its own structure (4Y9V), followed by hits to the O-specific polysaccharide lyases of *Pseudomonas* phages LKA1 (4RU4) and phi297 (4RU5) (Supplementary Table 8). The B9 and APK48 depolymerase models hit is to a poly(beta-d-mannuronate) c5 epimerase from *Azotobacter vinelandii* (5LW3 and 2PYH) and the putative pectin lyase gp18 (7CHU-A) of the *Geobacillus* virus E2. The TaPaz top hit is to the *E. coli* phage HK620 tail spike depolymerase (4XLA) and the poly(beta-d-mannuronate) c5 epimerase from *Azotobacter vinelandii* (5LW3). The top AbTj AlphaFold model DALI hit is to *Acinetobacter* phage phiAB6 tail spike depolymerase (5JSD), suggesting that this protein is responsible for the halo formation documented with the AbTj plaques. Similarly, the WCHABP1 depolymerase was modeled to a glycan biofilm modifying enzyme from *Pantoea stewartii* (6TGF), followed by a hit to the phiAB6 tail spike. These findings highlight a breakdown in the amino acid sequence of the proteins, but a conservation in structure, which has led to the missing functional domain hits of these proteins. The use of AlphaFold Multimer to model the proteins, and the DALI server for PDB search of the resulting models shows the power of these methods for investigating the function of tail fiber proteins lacking domain hits to investigate their potential functions.

## 4 Conclusion

Overall, this investigation into phage tail fiber domains has provided valuable insights into the diversity and functional characteristics of these proteins within the *Acinetobacter* phage. The domains associated with the depolymerase function were found to be pectin lyase-like (SSF51126), tail spike binding (cd20481), (trans)glycosidases (SSF51445), and potentially SGNH hydrolase. Furthermore, phage tail fibers with confirmed, or potential, depolymerase activity, but no functional domain hits, were modeled with AlphaFold Multimer and searched against the PDB database with the DALI server and hit to templates of other known depolymerase proteins, highlighting the power of this approach while investigating novel tail fiber proteins lacking functional domains. Although this study enhances our understanding, it is essential to recognize its limitations and the dynamic nature of scientific knowledge. Future research endeavors should further explore the role of these domains in phage-host interactions. This exploration will ensure a comprehensive grasp of their implications for phage therapy and bacterial pathogenesis.

## Data availability statement

The datasets presented in this study can be found in online repositories. The names of the repository/repositories and accession number(s) can be found in the article/Supplementary material.

## Author contributions

DP compiled raw data, performed analysis on the data, and wrote and edited the paper. WC edited the paper and acquired funding. FG analyzed the data and edited the paper. All authors contributed to the article and approved the submitted version.

## Supplementary material

Supplementary Table 1 Abbreviations used throughout the article.

Supplementary Table 2 Heatmap resulting from a Clustal Omega multisequence alignment of PLD containing proteins.

Supplementary Table 3 Heatmap resulting from a Clustal Omega multisequence alignment of Tailspike domain containing proteins.

Supplementary Table 4 Heatmap resulting from a Clustal Omega multisequence alignment of G3DSA containing proteins.

Supplementary Table 5 Heatmap resulting from a Clustal Omega multisequence alignment of SGNH hydrolase domain containing proteins.

Supplementary Table 6 Heatmap resulting from a Clustal Omega multisequence alignment of Galactose-binding domain containing proteins.

Supplementary Table 7 Heatmap resulting from a Clustal Omega multisequence alignment of lysozyme domain containing proteins.

Supplementary Table 8 Top five DALI hits from PDB90 for AlphaFold-modeled proteins.
